# Practical Observations on the Application of the Liquid Tests for the Detection of Arsenic and Corrosive Sublimate, When Administered as Poisons; and on the Preparations of the Contents of the Stomach, so as to Ensure the Characteristic Effects of the Decomposing Agents

**Published:** 1826-12

**Authors:** Robert Venables


### DETECTION OF POISONS.

*Practical Observations on the Application of the Liquid Tests for the Detection of Arsenic and Corrosive Sublimate, when administered as Poisons; and on the Preparations of the Contents of the Stomach, so as to ensure the characteristic Effects of the decomposing Agents.* By Robert Venables, M.B. &c. &c.*

In the application of a liquid test, with the view of identifying any agent in solution, two objects are essentially necessary to insure accuracy of research:—1. A precipitate must be formed, consisting (in part, at least,) of the substance, the presence of which we wish to identify. 2. We should always select such agents as will produce a coloured precipitate, and, if possible, one peculiarly distinctive of the substance

* We regret that press of matter has obliged us to postpone Dr. Venables' valuable paper longer than we could have wished.—Editor.

sought for. Hence it is that the soluble salts of silver and of copper, as forming (under particular management) coloured precipitates with arsenic, have been regarded as the agents most suited to the discovery of this mineral in solution. The precipitate formed by the combination of arsenic with the oxide of silver, is of a yellow colour; while the arsenite of copper is of a beautiful green.

When we have a suspected solution, we cannot at once pronounce on the absence of arsenic, if no precipitate occur on the simple addition of nitrate of silver, because the nitric acid exerts a stronger affinity for the oxide of silver than the arsenious acid. Hence we are recommended to add a small proportion of alkali, or alkaline carbonate, to the suspected solution, by which means we form an alkaline arsenite; in which case, on the addition of the nitrate of silver, a double decomposition takes place, and the characteristic precipitate is thrown down.

Several objections, however, have been urged against this test; and perhaps the most formidable, though the least insisted on, is that the nitrate of silver is decomposed both by the fixed alkalies and by their soluble salts, and the precipitates on these occasions are such as may readily deceive a careless observer, or one not habituated to the practical part of chemistry. Indeed, when nitrate of silver is decomposed by caustic or carbonated potash, the first addition produces a yellow blush, which very much resembles that of the arsenite of silver; and a hasty or inexperienced operator might, without waiting to examine the subsequent appearance, be induced to pronounce the presence of arsenic from the above appearance, however transitory.

Another objection is, that nitrate of silver produces, with the alkaline phosphates, precipitates which, in their sensible characters, so completely resemble the arsenite of silver as not to be distinguishable. It may be still further objected, that the alkaline muriates,—as common salt, for instance, and which is so likely to constitute a portion of the contents of the stomach,—throws down a copious white precipitate with nitrate of silver, which will tend to embarrass the operator, and certainly to obscure the results.

Various plans of obviating these objections have been proposed. Perhaps the most simple plan would be to apply the nitrate of silver to the suspected solution, previously to adding the alkali. If no precipitation take place, we naturally conclude that the fluid does not hold in solution either alkaline phosphates or muriates. Then we may add a drop of caustic or carbonated alkali in solution; when, if arsenic be

1

present, the characteristic yellow-coloured precipitate will immediately fall down. If, on the addition of the nitrate of silver, a copious, dense, white precipitate fall down, we may conclude this to be muriate of silver; and we should therefore cautiously add the nitrate of silver, till precipitation ceases. Then, by filtration, we remove the muriate of silver, and afterwards proceed as before. The objection, however, which was urged in the beginning,—namely, that the first precipitation from adding an alkali to nitrate of silver may deceive, or at all events render an inexperienced or unpractised operator uncertain as to the results, will still apply. In order to obviate these inconveniences, I would recommend the operator to test the suspected solution, in the first instance, for the two salts—muriate and phosphate of soda, which are most likely to embarrass his future proceeding. This may be effected in the following manner:—A drop or two of the suspected fluid may be placed on a card or white plate, and several of these may be so prepared. To one we may add nitrate of silver, which will discover the alkaline muriates, if they be present; and the sulphate or nitrate of iron, or the nitrate of lead, will discover the phosphate of soda.*

If, on the addition of these reagents, we discover either of the above salts in the solution, we should proceed to remove them by the cautious addition of their respective precipitants, and the subsequent filtration of the liquor. In this manner we may free the suspected fluid from all embarrassment, and every doubt and uncertainty arising from the above sources.

Less operose methods have been proposed for obviating these sources of fallacy in the application of the nitrate of silver. Dr. PARIS proposes the following:—"Drop (he says) the suspected fluid on a piece of white paper, making with it a broad line: along this line a stick of lunar caustic is to be slowly drawn several times successively, when a streak is produced, of a colour resembling that known by the name of Indian yellow; and this is equally produced by the presence of arsenic and that of an alkaline phosphate: but the one from the former is rough, curdy, and flocculent, as if effected by a crayon; that from the latter is homogeneous and uniform, resembling a water-colour laid smoothly on with a brush. But a more important and distinctive peculiarity soon succeeds; for in less than two minutes the phosphoric yellow fades into a sad green, and becomes gradually darker,

* The soluble salts of iron are preferable to those of lead for the detection of phosphate of soda, because the phosphate of iron is of a blue colour, while that of lead is white, which therefore is not so characteristic.

and ultimately quite black; while, on the other hand, the arsenical yellow remains permanent, or nearly so, for some time, when it becomes brown."* This statement is unquestionably correct, as I have verified its accuracy by actual experiment; but it is not so completely applicable under every variety of circumstance, as, from the observations of Dr. Paris, we might be led to imagine. We must observe, that it is merely capable of distinguishing between two fluids, the one holding arsenic and the other an alkaline phosphate in solution; and it enables us to identify them. But it may happen that the suspected liquor may contain both phosphate of soda and arsenic, and almost certainly muriate of soda. In these cases the indications will be very uncertain, and undoubtedly such as should not be relied on by unpractised operators.

Dr. Marcet suggested the superior efficacy of ammonia for the purposes of the double decomposition. This suggestion led Mr. Hume, the original proposer of nitrate of silver as a test for arsenic, to form an ammoniuret of silver at once, by throwing down the oxide of silver by adding ammonia to the solution of the nitrate,† and then redissolving nearly the whole of the precipitate by a further addition of ammonia. A portion of the precipitated oxide should be left, to prevent an over-excess of ammonia, which would prevent the precipitation of the arsenite of silver. Dr. Paris observes upon this preparation:—"This is an improvement of considerable value; for, while it obviates the necessity of ascertaining the exact proportion of alkali required in each experiment, it possesses the desirable property of not disturbing the solution of phosphate of soda."‡

With the former part of this statement I perfectly coincide; but I must beg leave to say, that the latter will not be war-

* Pharmacologia, art. Arsenic; and Med. Jurisprudence, vol.ii. p. 241, 242.

† I am at a loss to understand the following passage in the Pharmacologia and Medical Jurisprudence:—"And for this purpose Dr. Marcet suggested the superior advantages which would attend the application of ammonia, in all those cases where the arsenic had not been previously combined with a fixed alkali; since the former,* when added singly, does not decompose nitrate of silver." Hence we should infer that ammonia will not disturb nitrate of silver; but, in the directions for the preparation of an ammoniuret of silver, which immediately follow, we are desired to dissolve "ten grains of lunar caustic in ten times its weight of distilled water; to this to add, guttatim, liquid ammonia, until a precipitate is formed."—The truth is, that the addition of ammonia to nitrate of silver in solution, immediately throws down the oxide of the metal. When, however, the quantity of ammonia exceeds the equivalent for saturating the nitric acid, it begins to redissolve the precipitated oxide.

‡ Pharm. *ubi supra*; Jurisprudence, vol. ii. p. 244-5.

(* Ammonia.)

ranted by an appeal to experiment. The ammoniuret of silver throws down copious yellowish-white precipitates, with the solutions both of phosphate and muriate of soda; and, although the practised operator is in no danger of mistaking these colours for those of arsenic, yet the above observation, from such high authority, may betray an inexperienced investigator into a fatal mistake. Such are the results of my investigations into the validity of the nitrate of silver as a reagent for the detection and identification of arsenic; and it is from these objections, which suggested themselves during my experiments, that I am induced to recommend precipitating those salts, which are likely to confuse our examinations, if they should be found to be present.

The other reagent in most repute is the solution of copper. The arsenious acid forms with oxide of copper an insoluble salt, of a beautiful green colour, named, from its discoverer, "Scheele's green." The sulphate of copper is the salt generally used for the purpose under consideration. In this case also we are obliged to resort to double decomposition; for the other acids exert a stronger affinity towards copper than the arsenious. Dr. Bostock specifies the exact proportions of arsenic, sulphate of copper, alkali, and water, capable of producing the most conspicuous effect. This information must be regarded as matter of curiosity, rather than of practical utility to the purposes under review. We commonly meet with the arsenic dissolved, or we are obliged to dissolve it, in order to effect its separation from substances with which it may be intermixed, and the presence of which would tend to embarrass and confuse our experiments. In such cases it is evident that we can form no estimate of the quantity of arsenic, so as to enable us to apportion the other agents, and adjust the whole in their relative proportions.

Mr. Hume has proposed the ammoniuret of copper for the purpose of detecting and identifying arsenic in solution. This may be formed by adding ammonia to any of the soluble salts of copper,—as the sulphate, nitrate,* acetate, &c. and continuing the addition till nearly the whole of the precipitate is redissolved. The solution thus prepared assumes a beautiful deep-blue colour, which is changed to a green when added to any fluid holding arsenic in solution. The transition from deep-blue to green is extremely evident and satisfactory: add to which, that these preparations are not affected by the alkaline phosphates or muriates. This assertion is not the result of theory, but of actual experiment.

* I have some reason to believe the nitrate the preferable salt for this purpose.

I added the ammoniuret of copper, prepared from the sulphate, to a strong solution of phosphate of soda, and no precipitation whatever took place. I tried the same experiment with the ammoniuret prepared from the nitrate and deutacetate, and with the same results. But, on adding a solution of arsenic to each of these three fluids, the green colour was immediately evolved.*

It adds much to the certainty to perform these experiments upon a piece of white card, a tile, or white plate: the greenish precipitate is immediately deposited, and becomes sufficiently evident for thorough conviction. A drop or two is quite sufficient for the purpose. Hence it may be inferred that, as far as we are at present acquainted, the ammoniuret of copper is the most certain, as being subject to the fewest sources of fallacy.†

It may now be inquired, after having applied our reagents, and finding no traces, are we to conclude that arsenic does not exist in the suspected fluid? This question may be fairly replied to in the negative. I diluted a solution of arsenic till the addition of the most delicate reagents afforded no sensible indication of its presence. But, upon distilling the mixture when the fluid became concentrated, the action of the test became sensible. Hence, in all suspicious cases, it would be well to concentrate the suspected fluid by distillation or evaporation.

It will frequently happen that, when a fluid has been evaporated nearly to dryness, by continuing the heat, if the process have been performed by distillation, or applying a sufficient temperature, if otherwise,—that the arsenic will sublime, and may thus be collected and treated by the usual reagents.

I shall now proceed to offer a few observations upon the detection and identification of Corrosive Sublimate.

Various means have been devised for this purpose: for an account of them, I refer the reader to Paris's Pharmacologia and Medical Jurisprudence. I shall, therefore, confine myself upon this question to a process which has occurred to me, but which I do not find noticed in the systematic works. The habitudes of mercury with iodine afford ample and satisfactory means for its identification. If a fluid hold corrosive sublimate in solution, provided the solvent be not a spirituous

* The ammoniuret of silver, treated in the same manner, threw down dense copious precipitates, which obscured the results, and were by no means characteristic.

† Tannin, in a weak solution of arsenic, will obscure the action of the ammoniuret of copper; but this source of fallacy will be considered hereafter.

one, immediately on the addition of a dilute* solution of hydriodate potass, a yellowish cloudy appearance occurs, which soon assumes a beautiful red colour. This is an extremely delicate test; but, that we may obviate every source of fallacy, we should take care to distil off all those menstruums, spirits, essential oils, &c. in which the precipitate is soluble, and which might hence obscure its indications. The red precipitate, which is thus formed, exhibits distinctive characters, as its solubility in alcohol, &c. and in an excess of the precipitating agent, hydriodate of potash. Exposed to a moderate heat, it passes to a yellowish white colour, and iodine in vapour is seen to rise; and, lastly, the whole evaporates. Triturated with zinc or iron filings, it is decomposed, and metallic globules of quicksilver may be readily distinguished, intermixed with the mass. The best mode of exhibiting this phenomenon is to introduce the mixture, moistened with water, into a glass tube or the bowl of a tobacco-pipe, and urge it gently with the blow-pipe. Let the contents, when dry, be thrown out upon paper, and the metallic globules may be seen very distinctly; even though the quantities subjected to experiment may have been very minute.†

Corrosive sublimate is very readily decomposed; and therefore, although this poison may have been administered, the tests will afford no indication of its presence, in consequence of the partial reduction of the sublimate, and its conversion into calomel. Indeed, the means usually resorted to for relief, generally effect this reduction. Calomel, however, it should be recollected, is insoluble, while at the same time it is highly volatile, and easily sublimed by heat. In such cases, the solid contents of the stomach should be exposed to heat in a proper vessel, and the sublimed powder triturated with hydriodate of potass; when, if the powder be calomel, a greenish-yellow colour is produced, owing to the double decomposition which ensues. The resulting protiodide may be decomposed as above, and metallic mercury evolved.

Hitherto we have been supposing the most simple case, —viz. that the suspected fluids are not only transparent, but colourless. However, it must be obvious that, in cases of poisoning, we may expect to be embarrassed both by the

* It should be dilute, as the periodide is soluble in an excess of the hydriodate. It is also soluble in spirituous menstruums and essential oils.

† Hydriodate is a more preferable means of distinguishing corrosive sublimate from arsenic, than the means recommended by Brugnatelli, which is not only complicated, but unsatisfactory.

opacity and the colour of the vegetable and animal contents of the stomach. To obviate these sources of confusion, various ingenious methods have been proposed.

The opacity of the fluid may generally be removed by passing it through a common filter. By this means the substances mechanically suspended, or intermixed with the fluid, will be retained, and it will become transparent. But, if the opacity depend upon (as is often the case with the colouring principles,) matter soluble in the fluid, then other means must be adopted.

Mr. Philips proposes boiling the fluid with animal charcoal. The powers of the charcoal, however, in decolourising fluids, appear to me to be purely mechanical, and that it only acts as a very fine filter. It is, I believe, generally imagined that animal charcoal destroys vegetable and animal colouring matter by some chemical agency, which it exerts over these principles. Some experiments, however, which I instituted for determining the question, have induced me to adopt a different view, and to refer its effects entirely to mechanical principles.

*Experiment* 1.—I passed some infusion of litmus through ivory black, placed on a filter. The fluid passed through rapidly, but the colour was not so deep. The same fluid was returned, and passed through again; and this was repeated several times in succession. On each successive filtration, the infusion was longer in passing through, and there seemed to be less colouring matter. At last it appeared almost colourless; but, on adding a drop or two of sulphuric acid, a vivid red colour was immediately evolved.

2. The same phenomena followed on boiling, only the red colour was rather weaker.

3. Tea, with sugar and milk, were nearly discoloured by repeated filtration. By boiling, the colour was rendered less sensible.

4. Tincture of myrrh passed nearly unaltered; nor had boiling a more sensible effect.

5. A mixture of tincture of myrrh with water, in equal parts, was passed through. On adding water to tincture of myrrh, a portion of the resin is set at liberty by the solution, and was, of course, separated by passing through the filter. However, the colouring matter could not be wholly removed, even by boiling with ivory-black; although the evaporation of the alcohol, consequent on boiling, greatly reduced the quantity of colouring matter held in solution by the alcohol.

6. Several vegetable infusions, as senna, cascarilla, rhubarb, bark, jalap, quassia, &c. were similarly treated; but very little effect was made upon the colour of the fluid by these means. Indeed, passing them through fine sand had very nearly the same effect in reducing the quantity of colouring matter. Tea, soap-suds,* &c. were rendered nearly equally colourless, whether passed through sand or ivory-black; making allowance for the greater fineness of ivory-black, and of course its superior delicacy as a filter.

Certainly, I found that ebullition with ivory black considerably enhanced its powers. Thus, vegetable infusions generally came through less coloured (and frequently colourless) after boiling, than could be effected by any number of successive filtrations. I was almost inclined to attribute some kind of chemical agency to the ivory-black on observing this circumstance, although I could form no rational opinion whatever of its nature. But, upon reflection that long boiling renders some of the vegetable principles—extractive, for instance,—insoluble, it occurred to me that the removal of the colouring principle was owing to a similar cause, and that, in all probability, simple boiling would prove as effectual as boiling with animal charcoal: I therefore simply boiled these coloured fluids for some time, and found that it came through as little coloured as when ivory-black was boiled with it. Hence it may be concluded—

1st. The agency of ivory-black in decolourising fluids is purely mechanical, and that it acts merely as a delicate filter.

2. Boiling with this agent seems merely to precipitate the colouring matter, which is separated by the subsequent filtration.

3. When the colouring matter is chemically dissolved in the fluid, filtration through animal charcoal will not separate it: nor is it separable by boiling even, unless the process render the colouring matter insoluble,† when the agency of the charcoal is purely mechanical.

4. In mixed fluids, the colouring matter may be held in solution by a menstruum, the solvent powers of which may be weakened by dilution; or the menstruum may be volatile, and readily evaporated by a moderate elevation of temperature: in either case, the colouring matter may be precipitated, and afterwards separated by filtration.‡

* In soap-suds, the opacity and colour evidently depends upon clay, dust, grease, &c. mechanically suspended in the fluid. Filtering separates these; but soap in solution was readily detected in the filtered liquid, by adding a few drops of acid when the oil was set at liberty.

† We must not forget that boiling reduces the quantity of the solvent.

‡ I have not considered it necessary to adduce experimental proof of the inefficacy of ivory-black in preventing the passage of substances held chemically in solution.

Having, therefore, fully established that the action of ivory-black, in separating colouring matter, is purely mechanical, it may now be inquired what is the best mode of managing it so as to secure its full effects. From what has been already established, it is evident that boiling the coloured fluid with the charcoal is unnecessary; but it has been ascertained above, that colouring matter, which passed through on the first or second attempt, is separated by repeated filtration. This appears to me to arise from the porous texture of the mass, when in its dry pulverulent state. As, however, it becomes saturated with water or fluid, the particles are brought closer together,* and the mass becomes denser or more pasty. Hence the difficulty opposed to the passage of the minutest particles of colouring matter in a state of mechanical suspension. It has been remarked by Mr. Phillips, that in some cases the ivory-black seemed to contain a muriate. That intended for a filter, therefore, should be boiled in distilled water, and then washed with boiling distilled water, till the washings cease to precipitate muriate of silver on the application of lunar caustic. After these repeated washings (which should be performed under any circumstances), the ivory-black forms a dense solid cake, of a pasty consistence. A filtering paper, of a conical form to fit into a funnel, should now be formed, and the internal surface of this filter should be lined with the charcoal paste; and this lining should be spread pretty thickly up to the top of the filter, for otherwise the colouring matter may pass through the upper part of the paper, where it is not defended by the charcoal coating. Matters being thus arranged, the coloured fluid may be cautiously poured into the filter, and allowed to ooze through, which it will do but very slowly. If the filtered fluid should still be coloured, it may now be boiled with a little ivory-black, in a Florence flask, or any other convenient apparatus, and again passed through the filter, when, in all probability, after two or three successive filtrations, the fluid will pass through colourless.

If a solution of any salt be passed through or boiled with ivory-black, it may still be recognised afterwards by the application of the appropriate reagents. The salts of copper are not only recognisable, but the filtered fluid exhibits the characteristic colour.

* This is the principle upon which brewers return the first flowings from the mash-tub. When the wort first passes, it is quite thick, owing to the finer particles of the malt escaping: these flowings are returned repeatedly, till they come through quite fine. By the returning, the grains are caked about the cradle into a dense paste, which intercepts the firm particles of the matter, and prevents their escape.

It has been already observed, that, when the colouring matter is chemically dissolved in the fluid, this process will not answer. In cases of poisoning, the only species of colouring matter that we can expect to interrupt our proceedings must be of vegetable or animal origin. Those from the mineral kingdom generally arise from the exhibition of emetics, under the direction of the practitioner. Sulphate of copper being the only agent likely to interrupt the process, the practitioner will, of course, know how to remove it, if he should have prescribed it as an emetic. The colouring matter from the other sources, I believe, will seldom be found to resist the action of chlorine. If the processes above described should prove ineffectual in removing the colouring matter, then the practitioner may proceed to pass a current of chlorine gas through the coloured fluid, which generally will be found effectual in destroying the agency of the colouring principles. After passing the gas through the fluid, it may be again passed through the filter. It will frequently happen that it will have a green tinge from the admixture of chlorine. Boiling the fluid, however, will expel the chlorine, and render the fluid perfectly colourless.* If the reagents usually employed give no indications of arsenic, then the whole of the tested portions should be evaporated, when frequently, during the concentration, the arsenical precipitate will become apparent.

With the view of ascertaining the efficacy of this plan, I formed the most heterogeneous mixture I could devise, consisting of wine, beer, tea, soap-suds, infusions of senna, quassia, bark, with other tinctures, and divided the mixture into two equal parts. To one part a quantity of arsenic was added, while the other received no such addition. Both portions were treated separately, according to the process above mentioned for removing the colouring matter. Both were rendered nearly colourless, and, on applying the tests to portions of each, the one containing the arsenic gave evident indications of its presence; while no such result followed the application of the tests to the portion which contained no arsenic.

It may here certainly be objected, that the above, though curious, are not precisely the circumstances under which

* It should be recollected that these repeated boilings will tend to evaporate the watery part, and thus some arsenic may be precipitated. To obviate this, distilled water should be added, so as to keep up the original quantity of the solvent. If no traces of arsenic can be discovered, then the filtering paste should be exposed to heat with potash, when possibly metallic arsenic will sublime.

arsenic is presented to us in the human stomach, in cases of poisoning. In order to meet this objection, I endeavoured to bring the circumstances of the case to resemble that of poisoning as nearly as possible. For this purpose, a hearty dinner, consisting of fish, soup, meat (fresh and salt), with a due proportion of bread, potatoes, vegetables, salad, &c. was eaten; after which pastry, with cheese, was eaten, and a tolerable quantity of beer drank; three glasses of white and three glasses of port wine were taken, and after dinner a little fruit, with a glass of brandy and water, was taken. A cup of coffee, and one of strong tea, with sugar and milk, were the last things swallowed.

Under these circumstances, ipecacuanha was administered in twelve-grain doses, till the stomach was excited to eject its contents, which occurred after the exhibition of the third dose. The contents of the stomach was received into a basin, in which five grains of arsenic had been previously placed. The whole was now boiled with nearly a pint* of distilled water. The liquor was then passed through filtering paper, but the colour and consistence of the filtered fluid was such as to prohibit the application of tests, with any prospect of success. It was therefore boiled with ivory-black, passed through a filter prepared as above directed, and the remaining portion of the pint of distilled water, in a state of ebullition, poured on, so as completely to dissolve any portion of the arsenic which might have escaped solution. A current of chlorine gas was now passed through the filtered liquor, till it exhibited little else than the green colour characterising chlorine gas in solution, all the impurities having subsided. The admixture with chlorine was now again passed through the charcoal filter, by which all the sediment that had subsided was separated, and the fluid was perfectly transparent, though of a greenish colour. It was now boiled, by which the chlorine was expelled, and the fluid became perfectly transparent and colourless.† The application of the various reagents to separate the portions, gave the most decided and satisfactory indications of the presence of arsenic.

I feel almost perfectly assured that the above mode of proceeding will be found adequate to the detection of arsenic in every possible case; and it should be borne in mind that the means of discovering this poison in the humid way should be rendered subsidiary to its metallisation. The collection of

* f℥ xij. by admeasurement.

† If any sediment should appear on boiling to expel the chlorine, it may be filtered, when it will be removed.

the precipitates,* and their subsequent decomposition by black flux, should never be neglected.

It is evident that the various means of discovering and identifying arsenic may be had recourse to, by dividing the fluid into as many portions as we wish to institute experiments; and, in a case of such importance, these should be as numerous and diversified as possible. I would recommend selecting a portion for evaporation to dryness.† From a portion of the residue, the arsenic may be sublimed; and the arsenic remaining in the other portion, heated in a glass tube with a few grains of pulverised nitre, will immediately evolve nitrous gas; and, by adding water, arseniate of potass will be recognised on the application of lunar caustic, or nitrate of silver in solution. The sublimed arsenic may be similarly treated.

I have been actively engaged in the above investigation now nearly four months. Whoever is at all acquainted with the intricacies of the subject, and the various and diversified inquiries which may present in cases of poisoning, will at once admit the difficulties which may occur and tend to embarrass the medical jurist. Yet it is upon record, and indeed we frequently meet with instances of persons undertaking to solve the problem of poisoning, who certainly manifest as little competence to this task as to any other which possibly could be proposed to them. I should therefore recommend the practitioner who is not habituated to chemical manipulations,‡ to apply to those more practised than himself. Some, perhaps, may consider it a reproach not to be capable of the operative solution of such chemical problems; but I would remind the practitioner, that he may be fully competent to the practical duties of his profession, without being an operative chemist. If the physician is theoretically acquainted with the laws of chemical action, and with the reactions which chemical agents mutually exert on each other, he will never be betrayed into those blunders of prescription which but too often stand as a record of the ignorance of the prescriber,§ and a monument of opprobrium to his profession.

* After a great number of trials, I am inclined to think that the precipitation of arsenic from its solution by a current of sulphuretted hydrogen gas, and the subsequent reduction of the resulting sulphuret by treating it with potass, is the most certain and easily conducted process.

† This should be done at a temperature below 380° Fahr., otherwise the arsenic may be volatilised, and escape.

‡ It may be imagined that any one initiated or conversant with the theory of chemical action, is capable of undertaking the operative part. Let those who indulge in this opinion undertake the operative solution of some chemical problem, and they will soon be convinced of their error.

§ Vide Paris's Pharmacologia, *passim*.

To such I do not address myself, but to men capable of the practical duties of the profession, and to whom it certainly cannot be objected, as matter of reproach, that they are not thoroughly practised in all the more delicate manipulations of operative chemistry.

*Henley upon Thames; 1[illegible]th August,* 1826.

P.S.—We are generally told to test the suspected fluids in wine-glasses: these vessels are too wide, and thus tend to render the phenomena less sensible. Long, narrow one or two ounce phials, as recommended by Dr. Prout for examining the morbid states of the urine, I conceive much preferable; and the changes induced by the application of tests to chemical agents in such vessels, are much more easily observed.

---

